# Molecular weight distribution of raw and catalytic fast pyrolysis oils: comparison of analytical methodologies†

**DOI:** 10.1039/c9ra09726k

**Published:** 2020-01-22

**Authors:** Anne E. Harman-Ware, Kellene Orton, Chris Deng, Sophia Kenrick, Daniel Carpenter, Jack R. Ferrell

**Affiliations:** Biosciences Center, National Renewable Energy Laboratory Golden CO USA; National Bioenergy Center, National Renewable Energy Laboratory Golden CO USA jack.ferrell@nrel.gov; Wyatt Technology Corporation Goleta CA USA

## Abstract

Comprehensive analysis of the molecular weight distribution of raw and catalytic fast pyrolysis oils derived from biomass remains a key technical hurdle to understanding oil quality as it relates to downstream use and multiple methods may be necessary to accurately represent all components present. Here, we report the molecular weight distribution metrics of fast pyrolysis (FP) and catalytic fast pyrolysis (CFP) oils as determined by gel permeation chromatography (GPC) combined with UV-diode array (UV), differential refractive index (RI), and multi-angle laser light scattering (MALS) detection. The measured molar mass distributions revealed that FP oil consisted of a higher proportion of larger products relative to the low molecular weight products contained in the CFP oil. GPC/RI and UV methods showed FP oil to have higher weight-average molecular weight (*M*_w_) and number-average molecular weight (*M*_n_) than CFP oil based on elution time. However, GPC/MALS, determined the two oils to have similar overall molecular weight distribution metrics (*M*_w_ and *M*_n_) and yielded values significantly higher than those determined by RI and UV detectors relative to external standards. Overall, the use of a multiple detection GPC method could enable a more accurate comparison and determination of true molecular weight metrics of bio-oils.

## Abstract 2

Comprehensive analysis of the molecular weight distribution of raw and catalytic fast pyrolysis oils remains a key technical hurdle to understanding oil quality and multiple methods may be necessary to accurately represent all components present.

## Graphical abstract

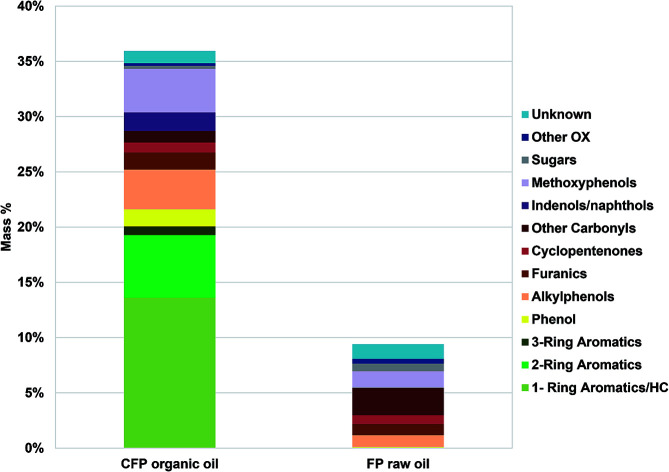

## Introduction

The thermal degradation of biomass by means of pyrolysis and catalytic fast pyrolysis processes has been the focus of extensive research for the production of bio-oils for use as fuels and to recover valuable coproducts.^1^ Many feedstocks, methods, reactor configurations, parameters and catalysts have been used to produce bio-oils of wide-ranging properties and composition.^2^ Additionally, different methods and associated parameters are used to analyze bio-oil properties and composition, many of which are based on standard analysis of petroleum-derived products and need to be tuned on the basis of oil properties or as such have not been standardized or have not been universally adopted.^3–5^ However, significant advancements have been made in the optimization of analytical methodologies for bio-oil analysis and subsequent understanding of the properties and composition of the bio-oils themselves. For example, NMR methods have been optimized to accurately capture functional group distribution in bio-oils from different processes.^6–8^ Solvent selection and extraction/fractionation processes have been explored to analyze different oil fractions particularly for NMR, GC/MS and molecular weight analyses by gel permeation chromatography (GPC).^4,9,10^ Various analytical methods have been used to attempt mass balance closure characterization of bio-oils;^11^ aqueous fractions have also been thoroughly characterized to account for the majority of products generated.^12^ Also, carbonyl content determination of bio-oils has become standardized using a titration method.^13–15^ Mass spectrometric analysis using FT-ICR MS,^16^ TOF,^17^ and standardized GC/MS methods^18,19^ have been used and optimized to characterize chemical classes and species of particular molecular weight/boiling point ranges of components of bio-oils.

As demonstrated particularly in mass spectrometric techniques, there is still need for the advancement of robust and adaptable analytical methodology for the determination of molecular weight distribution metrics (weight average molecular weight, *M*_w_, number average molecular weight, *M*_n_, *etc.*). The analysis of molecular weight distribution of biomass-derived bio-oils would provide information relating to the difficulties in employing upgrading strategies as well as the bulk properties of the oil relating to aging and stability and potential use as fuel.^20^ While many methods including mass spectrometry and GPC have been used to analyze molecular weight distribution of various types of pyrolysis oils (see review Harman-Ware *et al.* 2018 for details)^21^, there are few studies demonstrating the differences and similarities in results obtained from different analytical techniques using different parameters for different bio-oil types. Additionally, these authors are only aware of one study reporting on the use of size exclusion chromatography coupled with multi-angle laser light scattering (MALS) detection for the analysis of bio-oil components.^22^ The goal of this work was to determine the molecular weight distribution characteristics of FP oil and CFP bio-oils using GPC coupled with UV-DAD, RI detection and MALS detectors to understand the metrics on the basis of different analytical methodologies yielding information that was property-dependent.

## Methods and materials

### Bio-oil production

The biomass feed used to produce the oils was air-classified forest residues provided by Idaho National Laboratory and ground to <0.5 mm. The composition of the feedstock by ultimate analysis was 51.03 wt% C, 6.24 wt% H, 42.61 wt% O, 0.12 wt% N, <0.1 wt% S, and 0.41 wt% ash, and the moisture content was 1.75 wt%. Silica sand sized 300–500 μm from Black Lab, LLC (Chardon, OH) was used in the pyrolyzer as fluidizing media.

*Ex situ* catalytic pyrolysis was conducted in a dual fluidized bed reactor system^23^ using a ZSM-5 catalyst from Zeolyst (CBV 3024E), silica-to-alumina ratio of 30, sized 300–1000 μm after the original extrudates were ground and sieved. Biomass was fed into the pyrolysis reactor (5.2 cm inner diameter × 43 cm height) at a rate of 420 g h^−1^ over a 200 mL bed of silica sand. A cyclone separator was used to remove char from the stream allowing the pyrolysis vapors to enter the upgrading reactor (5.2 cm inner diameter × 15 cm tall lower section and a 7.8 cm diameter × 35.6 cm disengagement section). The ZSM-5 catalyst was metered into the upgrading reactor at a rate of 300 g h^−1^ and removed continuously *via* an overflow tube. A stainless-steel mesh hot gas filter was used to filter gases and upgraded vapors and then the vapors were condensed using various collectors including an air-cooled condenser, an electrostatic precipitator, dry-ice traps, and a coalescing filter. Nitrogen was used as the carrier gas during experiments at a flow of 17.4 L min^−1^ and total flow through the system was measured using a dry test meter. The pyrolysis temperature was 500 °C, while the upgrading temperature was 550 °C.

The fast pyrolysis oil was produced using the same system under the same conditions except the upgrading reactor was bypassed. The liquids from the condensation train receivers were combined and remained mixed for the fast pyrolysis experiment. CFP liquid products separated into three phases: top/light organic oil, middle aqueous liquid, and bottom/heavy organic oil. The fractions were separated by decanting, weighed, and analyzed separately for composition analyses. A weighted average CFP oil composition was then calculated based on the mass fraction of the light and heavy organic fractions. The two oil phases were recombined in representative fractions for GPC analysis to enable more direct comparison to FP oil analysis.

### Composition analysis of pyrolysis oils

The liquids were analyzed for elemental composition (C, H, N, O by difference) by combustion analysis on a LECO system and water content was determined by Karl Fisher titration. ^13^C NMR analysis was performed according to Happs *et al.*^6^ for functional group determination and GC/MS analysis of the oils was used to characterize specific components of the volatile and semi-volatile fractions of the oils. For GC/MS analysis oils were diluted 1 : 20 in acetone and 1 μL was injected into an Agilent G1530A GC-HP 5973 MS with a 30 m × 0.25 mm × 0.25 μm Restek Rtx-50 (50%-phenyl-methylpolysiloxane phase) column. The GC oven temperature was held at 40 °C for 2 minutes, ramped to 140 °C at 7 °C min^−1^, then to 290 °C at 12 °C min^−1^, and held for 5 minutes. The inlet temperature was 250 °C, transfer line temperature 300 °C, and helium carrier gas flow of 1 mL min^−1^ with a split ratio of 10 : 1. Calibration standards were used for external semi-quantification of several compounds detected in the samples (specific GC/MS calibration information is provided in ESI Table 1†).

### GPC

Bio-oil samples were solubilized at 1 mg mL^−1^ in tetrahydrofuran (THF). GPC analysis using RI and UV-DAD (or referred to as simply UV) was performed using an Agilent 1200 HPLC with 3 GPC columns (Agilent, 300 × 7.5 mm) packed with 10 μm polystyrene-divinyl benzene copolymer gel beads with nominal pore diameters of 10^4^, 10^3^ and 50 Å. The UV-DAD measured absorbance at 270 nm (80 nm bandwidth). An injection volume of 20 μL was used with an eluent (THF) flow rate of 1.0 mL min^−1^ for a total run time of 45 min. Polystyrene standards (Agilent Technologies) were used to calibrate for relative *M*_w_ and *M*_n_, with assumptions of Mark–Houwink parameters for relative comparison only. GPC analysis using MALS coupled with differential RI and viscometry analysis was performed using the same chromatographic method and columns described for UV detection. A Wyatt DAWN HELEOS II detector with a 785 nm MALS was used with a Wyatt ViscoStar III viscometer and Wyatt Optilab T-rEX differential refractometer (RI) with 785 nm LED. Data were collected and processed using ASTRA 7 for molecular weight analysis.

## Results and discussion

### Bio-oil composition differences

**Fig. 1:**
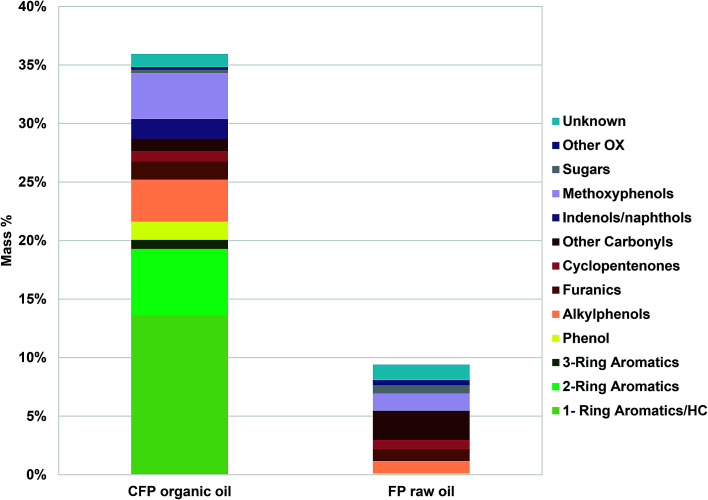
Composition (wt%) of pyrolysis oils determined by GC/MS. Other OX refers to oxygenates not categorized otherwise.

Bio-oil compositional properties obtained from compositional analysis, ^13^C NMR, viscometry and Karl Fischer analyses are outlined in Table 1. The CFP oil has lower oxygen content and higher carbon content in comparison to the FP oil. The reduction in oxygen content upon catalytic upgrading was due mostly to the reduction in carbonyl (C=O) and aliphatic C–O functionality, whereas aromatic C–O and methoxyl oxygen was similar in abundance between the FP and CFP oils. Aliphatic and aromatic C–C functionality was similar between the two oils but the CFP had significantly higher aromatic C–H content than the FP oil. Results from GC/MS analysis, Fig. 1, are relatively consistent with ^13^C NMR analysis even though GC/MS is only capable of characterizing approximately 36 wt% of the CFP oil and 10 wt% of the FP oil based solely on compounds that were calibrated for. The GC/MS results indicate that a significant portion of the oils, particularly of FP oil, was not capable of being analyzed by GC/MS. The lower abundance of analyzable species from FP oil in comparison to CFP oil is consistent with a greater abundance of acids (need derivatization for GC/MS analysis) and larger, high-molecular weight components (that do not volatilize, discussed in the following section) in the FP oil which also had a lower abundance of upgraded aromatic species capable of GC/MS analysis (1, 2, and 3-ring aromatics; *i.e.*, benzene, naphthalene). Differences in the composition between the two oils based on GC analysis also show CFP oil contains larger abundances of phenolics (including methoxyphenols, alkylphenols) as well as furan species but lower abundances of GC-analyzable sugars, other oxygenates and unknown compounds in comparison to the FP oil.

**Table 1:** Properties of FP and CFP oils obtained from ^13^C NMR and CHO analyses (% values reported on moisture free basis) as well as intrinsic viscosity, [*η*] determined by viscometry with standard deviation of triplicate analyses

Bio-oil	C=O (%)	Aromatic C–O (%)	Aromatic C–C (%)	Aromatic C–H (%)	Aliphatic C–O (%)	Methoxyl (%)	Aliphatic C–C (%)	C (%)	H (%)	O (%)	KF water (%)	Intrinsic viscosity (mL g^−1^)
Raw fast pyrolysis oil (FP)	13.8	13.3	11.8	20.5	20.6	2.6	17.4	58.6	6.7	34.6	17.3	2.99 (±0.04)
Catalytic fast pyrolysis oil (CFP)	6.7	14.8	13.5	42.1	4.1	1.9	17.1	75.4	7.0	17.3	4.8	2.42 (±0.05)

Refractive index increments (d*n*/d*c*) are important considerations for accurate molecular weight analyses using light scattering experiments and were determined to be 0.091 mL g^−1^ and 0.152 mL g^−1^ for the FP and CFP oils, respectively. The higher d*n*/d*c* values determined for the CFP oil are consistent with the presence of more polarizable functionalities^24^ present, which likely arises from the higher abundance of reduced species consisting of C–H (most of which are aromatic). Intrinsic viscosity, [*η*], was also slightly higher for CFP oil, being 2.99 mL g^−1^ in comparison to 2.42 mL g^−1^ determined for the FP oil and was relatively consistent throughout the samples (ESI Fig. 1†).

### Molecular weight distribution analysis by GPC

**Fig. 2:**
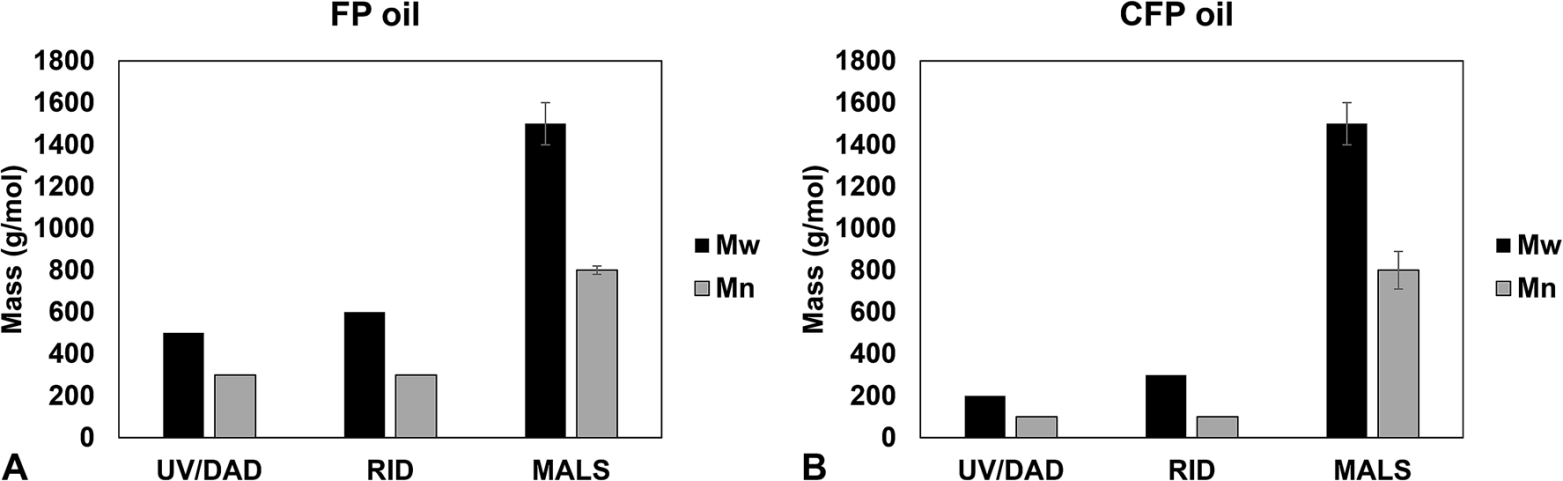
Molecular weight distribution metrics determined for (A) FP oil and (B) CFP oil measured using GPC with different detectors. Error bars indicate standard deviation for triplicate analysis, otherwise values were determined based on single injection analysis.

**Fig. 3:**
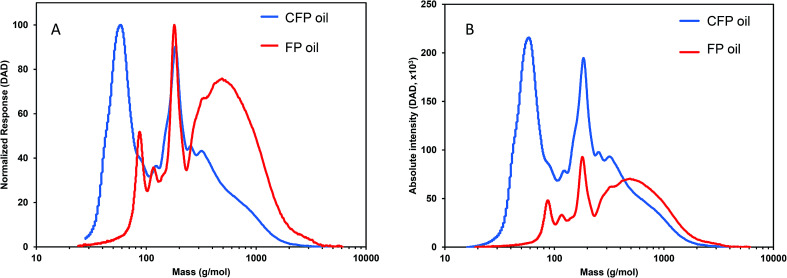
GPC-UV-DAD chromatograms from FP and CFP oils with (A) intensity normalized to maximum signal and (B) absolute intensity. DAD at 270 nm, oils 1 mg mL^−1^ in THF, elution volume converted to molar mass based on calibration to polystyrene standards.

**Fig. 4:**
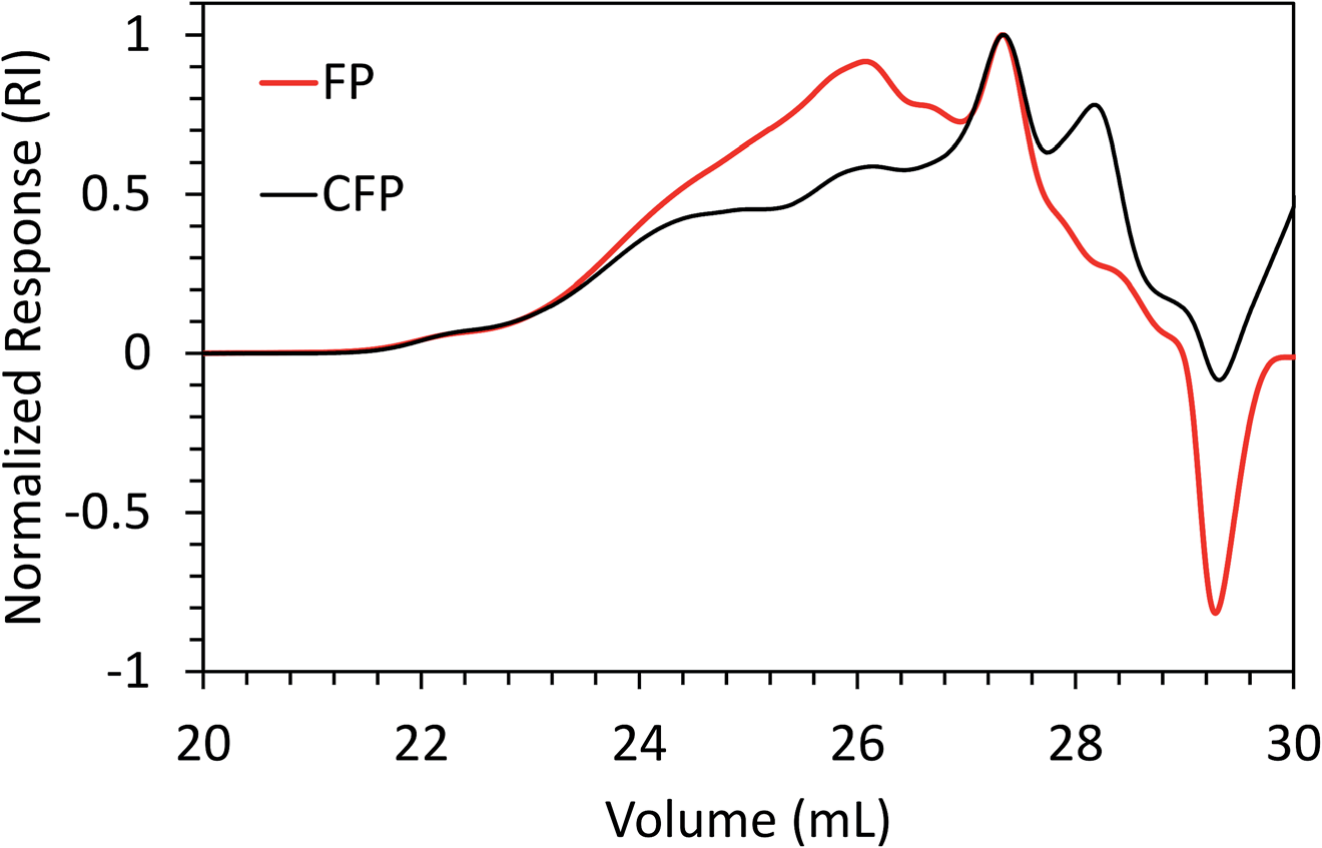
Overlay of GPC chromatograms from FP and CFP oils as detected from differential RI with signal normalized to maximum intensity.

Weight-average molecular weight (*M*_w_) and number-average molecular weight (*M*_n_) for FP and CFP oils for each different method of GPC detection are shown in Fig. 2. A similar *M*_w_ was determined using different UV and RI detectors relative to polystyrene (PS) for each FP and CFP oils, with both detectors consistently generating a higher *M*_w_ for the FP oils. Chromatograms shown in Fig. 3 and 4 for UV and differential RI detection, respectively, show that the CFP oil consists of higher abundances of smaller, lower molecular weight species and relatively lower abundances of the presumably larger, high molecular weight species. Fig. 3B, obtained from oils of the same dilution (1 mg mL^−1^ THF), shows higher intensities of the smaller sized, low molecular weight species and overall higher area of the CFP oil chromatogram, indicating an increased abundance of species (*i.e.*, phenolics, aromatics) that absorb UV at 270 nm. However, Fig. 3B makes it appear that the CFP oil consists of a larger abundance of species between ∼200–500 g mol^−1^ in comparison to the FP oil on concentration-fixed bases. Based on a comparison to MALS calculated distributions (discussed later), this could be partly due to the difference in the concentration and absorption of species with differing functionality eluting at those elution volumes. Consistent with UV detection, normalized GPC RI chromatograms in Fig. 4 also show CFP oil to contain lower abundances of high molecular weight species (larger molecules, molar masses > 500 g mol^−1^, lower elution volume) relative to the low molecular weight species, particularly those present in the permeation volume as well. Broader signal intensities from differential RI were observed which could likely be the result of UV detection at 270 nm not being sufficient to capture the range and intensity of species that RI detection observes. Additionally, differential RI detected low molecular weight species eluting in the permeation volume and they otherwise may not be accurately accounted for in molecular weight distribution calculations.

**Fig. 5:**
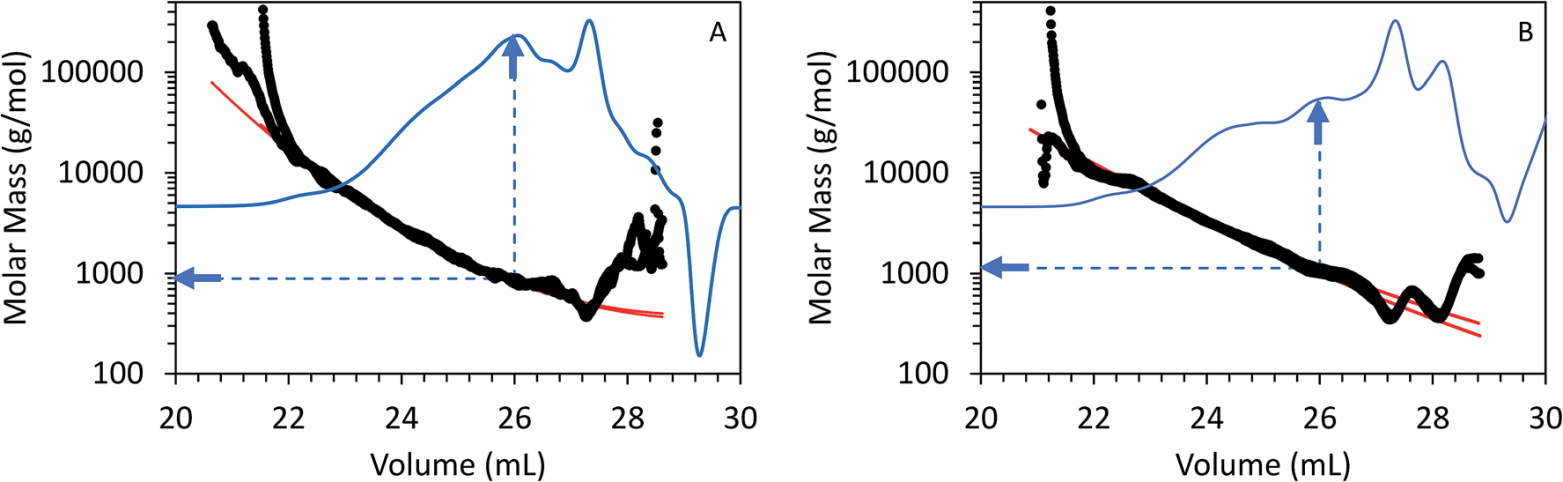
GPC chromatograms from (A) FP and (B) CFP oils as detected from differential RI with signal overlays of molar mass as determined by MALS. Arrows correspond to molar mass determined at elution volume of 26.0 mL for each bio-oil.

Fig. 5 shows triplicate differential RI chromatograms of the oils overlaid with the molar mass of each eluting species measured by MALS detection. Results fitting (Fig. 5, red line) was employed to extend the measurement accuracy in regions with low signal : noise ratio and where the dRI signal was confounded by the permeation volume. (UV-DAD data were collected simultaneously but not included in Fig. 5 for clarity). In comparison to RI or UV chromatograms where polystyrene standards were used to convert the elution volume to relative molar mass (RI chromatograms coupled with UV-DAD here not shown for redundancy were used to calculate molecular weight, dRI chromatograms coupled with MALs are displayed in Fig. 4 and 5), molar mass measured by MALS relies on the measured light scattering intensity and concentration, in this case by dRI, to determine an absolute molar mass at each data point across the chromatogram. Molar mass moments and distributions by MALS were larger than those estimated by elution time alone and similar to results reported by Ruiz *et al.*^22^ For example, the species eluting at approximately 26 mL in the chromatograms from both FP and CFP oils shown in Fig. 3A are determined to be approximately 500 g mol^−1^ based on their elution time, assuming the oils can be considered to have the same conformation and density as the polystyrene standard, whereas the corresponding molar masses were determined to be 800 g mol^−1^ based on MALS detection. While a 300 g mol^−1^ discrepancy would otherwise be negligible for large polymer applications, it is significant in the context of bio-oils consisting primarily of species <10 000 g mol^−1^. Additionally, the weight fraction distributions determined from dRI-MALS for species >500 g mol^−1^ was approximately 44% in the FP oil and 35% in the CFP oil (ESI Fig. 2†). It is possible that MALS provides a more accurate analysis of the molar mass since it actually measures the mass at each elution volume, although some error could be attributed to deviations of d*n*/d*c* of species at different elution volumes. This is in contrast with measurements relative to polystyrene elution times assuming analytes are of similar properties, thereby enabling the application of conventional calibration from external polymer standards.^25,26^

Compositional and property differences between FP and CFP oils as well as how these oils vary from traditional standards used for GPC molecular weight calibration provided the basis of performing this study to further understand the properties of these oils and advance the development of standard protocols of analysis. Since the oils consist of components <10 000 g mol^−1^, primarily being aromatic, phenolic and aliphatic molecules of less than 500 g mol^−1^, GPC is largely used to identify the presence of larger, higher mass species (recalcitrant oligomers derived from sugars and lignin as well as polyaromatic and condensed products)^27^ relative to smaller, low molecular weight biopolymer-derived monomers and upgraded products.^21,23,27,28^ If oils of drastically different species, functional groups, and bulk properties are being compared and comprehensive analysis is inherent to the study, it would be pertinent to incorporate accurate property values in calculations and utilize relevant GPC detection techniques for comparison.

For example, oils with varying abundances of UV-absorbing species (here, functional group differences determined in advance on the basis of NMR) should not be compared using UV-DAD detection following GPC unless the analysis is interpreted to be specific for those species. Additionally, differences in d*n*/d*c* measurements obtained for different oils and their consistency throughout the samples are important, particularly in the context of light scattering experiments for obtaining true molecular weight values.^29^ With that said, any variation of d*n*/d*c* within the oil samples that changes with molar mass could impart error on the accurate measurement of molar masses of individual chromatographic fractions (for RI detection as well). Therefore, the separation and measurement of d*n*/d*c* of different molecular weight fractions could potentially provide more accurate molecular weight distributions for the different fractions of bio-oils. The measurement of intrinsic viscosity [*η*] also provides insight of property differences in addition to being necessary to understand results in the context of deriving results from polymer standards based on elution time (universal calibration method).^29^ Therefore, the ability to detect the full range of species and accurately measure the molecular weight of species eluting would make the use of multi-detection techniques optimal for comprehensive GPC analysis of thermochemically-derived oils. While accurately detecting and predicting molecular weight of analytes eluting near the permeation volume require fitting and could be associated with some error for any detector, MALS can provide accurate molecular weight metrics of larger species and, in conjunction with RI, UV, viscometry and external calibrants, the full range of species can be accounted for.

Overall, it appears that comparison of molecular weight metrics of FP to CFP oils could potentially be obtained by GPC-RI relative to standards as long as small molecules eluting in the permeation volume can be properly accounted for or are otherwise noted when reporting values and that RI detector is sufficiently sensitive to detect oil species at a given dilution. Additionally, comparisons of oils corresponding molecular weight values should be deemed relative to standards and not necessarily reported as accurate or absolute values as standards may not be representative of all species present in the oils. These findings are in agreement with previous reports suggesting it would be best to use GPC and molecular weight metrics determined using external calibrants as a tool for comparison, particularly of oils of similar or incrementally different properties, more than an accurate predictor of specific molecular weight values.^30^ However, improvements in GPC resolution and particularly the detection methodology coupled with determination of other oil properties as described herein, could potentially provide more meaningful comparisons of molecular weight distribution and values, especially of oils of very different properties and functionality. Future investigations on the development of a standardized analytical methodology for bio-oil molecular weight distribution measurments would therefore incorporate the following steps: (1) measurement of d*n*/d*c* of oils (2) measurement of intrinsic viscosity of oils, and (3) measurement of molecular weight metrics relative to standards with proper constants in calculations using GPC-RI. Further method development involving column and solvent selection may also be necessary and may depend on initial screening tests. If necessary, follow-up analysis, particularly of the heavy fractions using MALS or mass spectrometry-based methods can be used to more accurately capture true molecular weight (or molar masses) of those species present. Preparative-scale GPC could also be employed to separate fractions and analyze fractions using various methods to obtain true molecular weights of the species present and corresponding distribution values.

## Conclusions

This study demonstrates how differences in the composition and physical properties of raw and catalytically upgraded fast pyrolysis oils can impact molecular weight distribution measurements. In order to obtain accurate values and representative comparisons of thermochemically-derived oils, as well as develop standard protocols for analysis, oils need to be analyzed for specific composition and property differences and analytical techniques need to be adjusted and properly interpreted to account for these differences. Here, we have demonstrated the need to characterize the functional groups (NMR, GC/MS), intrinsic viscosity (viscometry) and refractive index increments (d*n*/d*c* dilution experiments in conjunction with RI) of oils to properly analyze and interpret the molecular weight metrics as determined by MALS, RI and UV detection from GPC. MALS detection after GPC is reported here for the first time in the analysis of CFP oils and provides the first comparison of pyrolysis oils here to show higher molecular weight distributions than the other detection methods. This difference may in part be due to the sensitivity of MALS for high molar mass species, relative to other techniques, and also to limitations in quantifying light scattering intensity and concentration for low molar mass species eluting near the column's permeation volume. UV detection is not likely accurate as it does not detect all species present in the oils. GPC-RI may provide sufficient comparison of molecular weight distributions of oils relative to standards and is likely the most readily available technique for implementing a common method for the broader community producing biomass-derived pyrolysis oils. Multiple detection techniques, particularly GPC coupled with RI, viscometry and MALS could potentially provide the most comprehensively accurate molecular weight distribution comparisons, particularly of heavy components, present in bio-oils.

## Conflicts of interest

The authors declare no conflicts of interest.

## Funding

This work was authored by the National Renewable Energy Laboratory, operated by Alliance for Sustainable Energy, LLC, for the U.S. Department of Energy (DOE) under Contract No. DE-AC36-08GO28308. Funding provided by the U.S. Department of Energy Office of Energy Efficiency and Renewable Energy Bioenergy Technologies Office. Funding was also provided by the DOE Office of Science, Office of Biological and Environmental Research through the Center for Bioenergy Innovation (CBI), a DOE Bioenergy Research Center. The views expressed in the article do not necessarily represent the views of the DOE or the U.S. Government. The U.S. Government retains and the publisher, by accepting the article for publication, acknowledges that the U.S. Government retains a nonexclusive, paid-up, irrevocable, worldwide license to publish or reproduce the published form of this work, or allow others to do so, for U.S. Government purposes.

## Supplementary Material

RA-010-C9RA09726K-s001
