# Supplementary material for: Molecular weight distribution of raw and catalytic fast pyrolysis oils: comparison of analytical methodologies
Source: RSC Adv. 2020 Jan 22;10(7):3789–95. doi: 10.1039/c9ra09726k (PMC9048943; doi:10.1039/c9ra09726k)
Supplement: RA-010-C9RA09726K-s001 [file RA-010-C9RA09726K-s001.pdf]

Electronic Supplementary Material (ESI) for RSC Advances.
This journal is © The Royal Society of Chemistry 2019

Supplementary Material for Molecular Weight Distribution of Raw and Catalytic Fast Pyrolysis Oils: Comparison of Analytical Methodologies

Harman-Ware et al.

Supplementary Table 1. GC/MS calibration standards and concentration ranges

Compound	Concentration (mg/mL)
Acetaldehyde	0.05 - 1.0
Benzene	0.05 - 1.0
n-octane	0.10 - 2.0
1-octene	0.05 - 1.0
cis-1,4-dimethyl-cyclohexane	0.10 - 2.0
Toluene	0.05 - 1.0
p-Xylene	0.10 - 2.0
n-decane	0.10 - 2.0
2-Cyclopentenone	0.05 - 1.0
Benzene, propyl-	0.05 - 1.0
Phenol	0.05 - 1.0
2,3- Benzofuran	0.05 - 1.0
Indene	0.05 - 1.0
o-Cresol	0.05 - 1.0
o-Methoxyphenol	0.05 - 1.0
Tetralin	0.10 - 2.0
Naphthalene	0.05 - 1.0
Phenanthrene	0.05 - 1.0

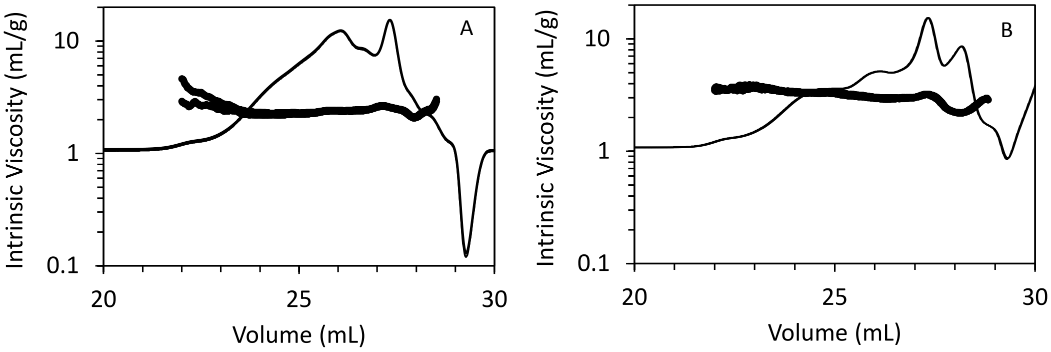

Supplemental Figure 1. Intrinsic viscosity, [η], as a function of elution volume for FP and CFP oils with reference to differential RI-derived chromatograms.

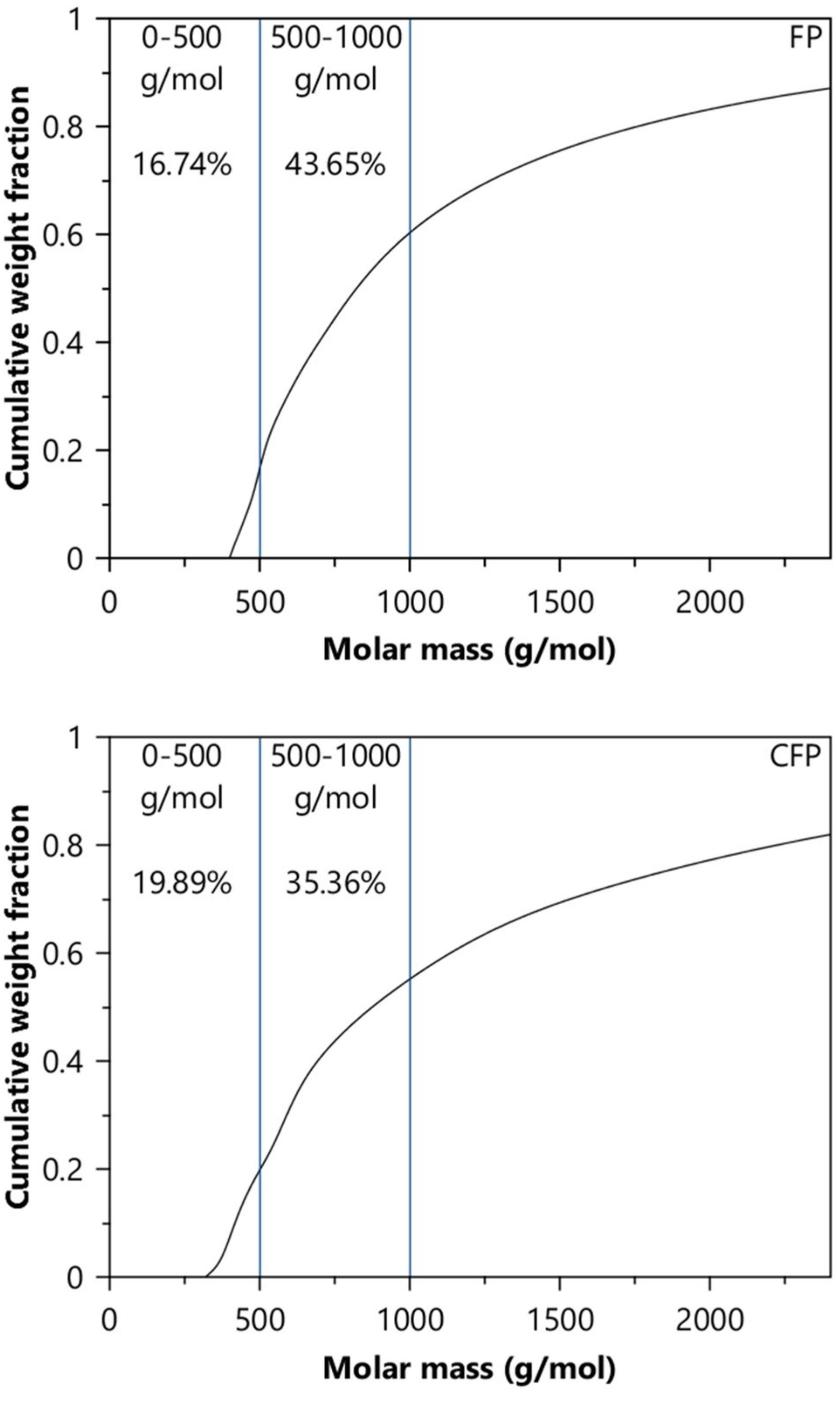

Supplemental Figure 2. Weight-fraction distributions measured by GPC show a lower distribution of analytes >500 g/mol in CFP oil (B) relative to FP oil (A). Analytes in permeation

volume not accounted for, hence the fraction of low mass species is underestimated, particularly for CFP oil.
